# Characterization of nanostructured ZnO thin films deposited through vacuum evaporation

**DOI:** 10.3762/bjnano.6.100

**Published:** 2015-04-16

**Authors:** Jose Alberto Alvarado, Arturo Maldonado, Héctor Juarez, Mauricio Pacio, Rene Perez

**Affiliations:** 1 Centro de Investigaciones y de Estudios Avanzados del Instituto Politecnico Nacional, programa de Nanociencias y Nanotecnología, Av. Instituto Politécnico Nacional # 2508, Col. San Pedro Zacatenco, C.P. 07360, México, D. F., México; 2 Centro de Investigaciones y de Estudios Avanzados del Instituto Politécnico Nacional, Sección de Electrónica del Estado Solido, Av. Instituto Politécnico Nacional # 2508, Col. San Pedro Zacatenco, C.P. 07360, México, D. F., México; 3 Centro de Investigaciones en Dispositivos Semiconductores, Benemérita Universidad Autónoma de Puebla, Ciudad Universitaria Avenida San Claudio y 14 Sur, 72570, Puebla, México

**Keywords:** nanostructure, thin film, transmittance, vacuum evaporation, X-ray diffraction (XRD)

## Abstract

This work presents a novel technique to deposit ZnO thin films through a metal vacuum evaporation technique using colloidal nanoparticles (average size of 30 nm), which were synthesized by our research group, as source. These thin films had a thickness between 45 and 123 nm as measured by profilometry. XRD patterns of the deposited thin films were obtained. According to the HRSEM micrographs worm-shaped nanostructures are observed in samples annealed at 600 °C and this characteristic disappears as the annealing temperature increases. The films obtained were annealed from 25 to 1000 °C, showing a gradual increase in transmittance spectra up to 85%. The optical band gaps obtained for these films are about 3.22 eV. The PL measurement shows an emission in the red and in the violet region and there is a correlation with the annealing process.

## Introduction

ZnO is a II–VI semiconductor compound that has been studied due its wide range of applications in nanodevices and nanosystems [1–2]. It has a wide band gap (3.37 eV), and a high exciton binding energy (60 meV) at room temperature, which allows for an efficient UV emission from the exciton, making it suitable for UV-emitting devices [3]. Depending on the form and the shape of the deposited thin films, they can be used in many applications, such as gas sensors [4].

A wide range of techniques to deposit thin films are used, such as molecular beam epitaxy (MBE) [5], single-source chemical vapor deposition (SS CVD) [6], metalorganic chemical vapor deposition (MOCVD) [7], sol–gel [8], spray pyrolysis [9], and RF magnetron sputtering [10]. However, the vacuum evaporation technique is used to deposit worm-form nanostructured thin films. This technique is different from those reported in the literature such as thermal evaporation assisted by inert gases [11], or e-beam evaporation [12]. So this technique was chosen because is easy to handle and only the deposition time is considered a control parameter. Another reason is the use of colloidal nanoparticles with well-controlled size, which were synthesized at the laboratory of the authors [13].

## Experimental

The proposed thin film deposition is a novel method to obtain nanostructures and thin films. This is an easy way to obtain two dimensional (2D) nanostructures using a simple vacuum system; the deposition is controlled just by varying the time of the source evaporation. The first step of this method is placing 0.5 g of nanocrystalline ZnO powder on a tungsten boat. These powders are resistively heated and sublimated from the tungsten boat, placed 2 cm below and parallel to the substrate. The evaporation chamber has a pressure work of 4 × 10^−4^ Torr, and the current applied to the resistor is 42 A which resulted in a sufficiently high temperature to sublimate the nanoparticles. Deposition times of 5, 10, 15, 20, and 25 min were chosen. After deposition, the substrate with the thin film is removed from the system and characterized for further analysis. These characterizations were carried out for un-annealed and annealed samples (from 25 to 1000 °C for 1 h in air), in order to observe the evolution in the formation of these 2D nanostructures.

Thin films were deposited using the vacuum metal evaporation system Jeol model JEE-420. The annealing process was done in a conventional oven to reorganize the crystallites and increase the adherence to the substrate. The structure of the 2D nanostructured thin films was characterized by X-ray diffraction (XRD) using a D8 Discover diffractometer for crystal phase identification. The morphology and shape of the ZnO nanostructured films were studied with a high-resolution scanning electron microscope (HRSEM) Carl Zeiss Auriga. The UV–vis spectra were obtained by using a Falcon evolution 600 spectrometer, the thickness was measured with a profilometer.

## Results and Discussion

### Structural properties of nanostructured thin films

**Figure 1:**
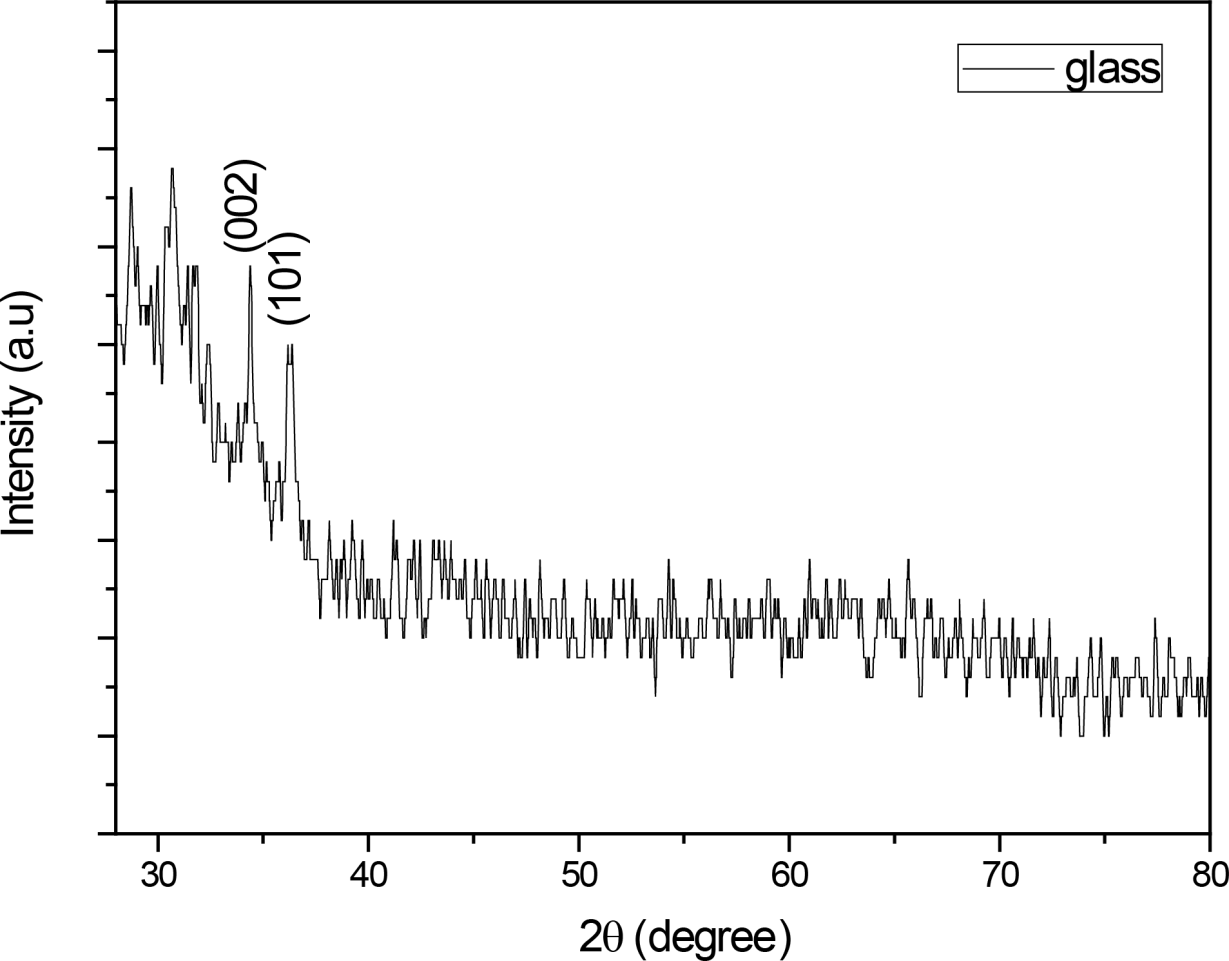
XRD pattern of nanostructured thin films on a glass substrate.

Figure 1 shows XRD pattern of the films deposited onto corning glass and silicon substrates annealed at 25, 200, 400, 600, 800 and 1000 °C in air. This result demonstrates that there are two peaks in the pattern at 34.4 and 36.2°, which corresponds with the (002) and (101) planes of ZnO, respectively. The weakness of the peaks is related to the thickness of the thin films. The peaks correspond directly to the hexagonal structure of the ZnO. This is due the nature of the source material, and it is assumed that only nanoparticles migration from the source to the substrate takes place.

### Morphology of the films

**Figure 2:**
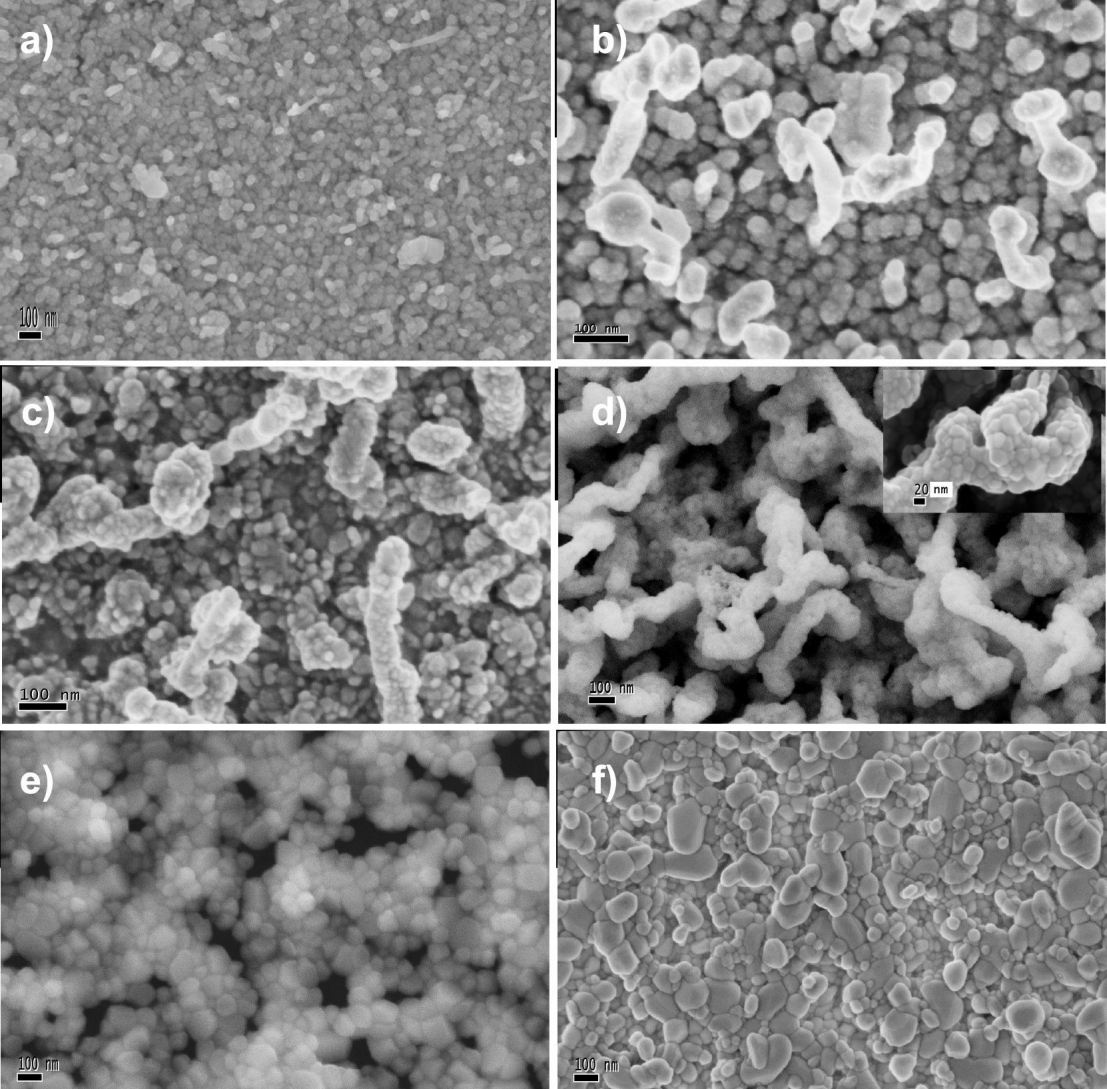
HRSEM micrographs of the thin films a) un-annealed, b) 200, c) 400, d) 600, e) 800 and f) 1000 °C annealing temperature.

Figure 2 shows the HRSEM pictures for a) un-annealed films, and films annealed at b) 200 °C, c) 400 °C, d) 600°C with a zoom in on the thin film, e) 800 °C and f) 1000 °C. From Figure 2a, it can be seen that there is a migration from the source to the substrate. It is hypothesized that the nanoparticles only decompose into particles small enough to cross the distance between the boat and glass substrate, this is supposed because the temperature in the resistor is not enough to decompose ZnO into ions. This decomposition takes place at 1500 °C and the resistor only reaches 800 °C. At these temperatures only migration takes place. However, this deposit is a mesh of particles that do not have an ordering, and thus the grain boundaries from each nanoparticle are not activated. Therefore, the adherence to the substrate is poor, which is also seen in the transmittance results. Varying the duration for which the films are annealed results in a perceptible physical change, and this is correlated to the reorganization of the particles in a preferential way, activating the boundaries of the nanoparticles. As shown in Figure 2d inset, the heat treatment provokes coalescence and the rapid formation of worm-shaped nanostructured thin films.

**Figure 3:**
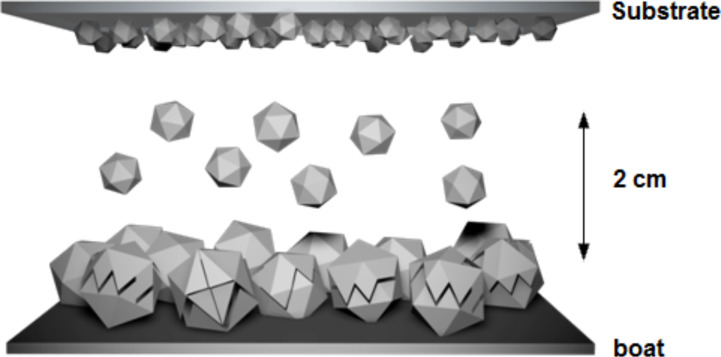
Nanoparticles migrate from source to substrate.

According to Figure 3, the authors propose a physical deposition or migration of small nanoparticles from the source supported of the resistor temperature, and not a total decomposition of the nanoparticles in ZnO–ZnO molecules, as was expected. The given nanoparticles of 30 nm in average size used as the source are decomposed into two or three smaller particles that migrate to the substrate. When this particle reaches the substrate, it lacks surface energy to be deposited firmly and has a C-axis orientation growth. However, when the surface energy is activated from an external source, it gives the system a rapid reorganization and coarsening, which permit the formation of the worm-shaped nanostructured thin films at 600 °C. This organization could be the cause of the increasing transparency of the thin films.

### Optical properties

**Figure 4:**
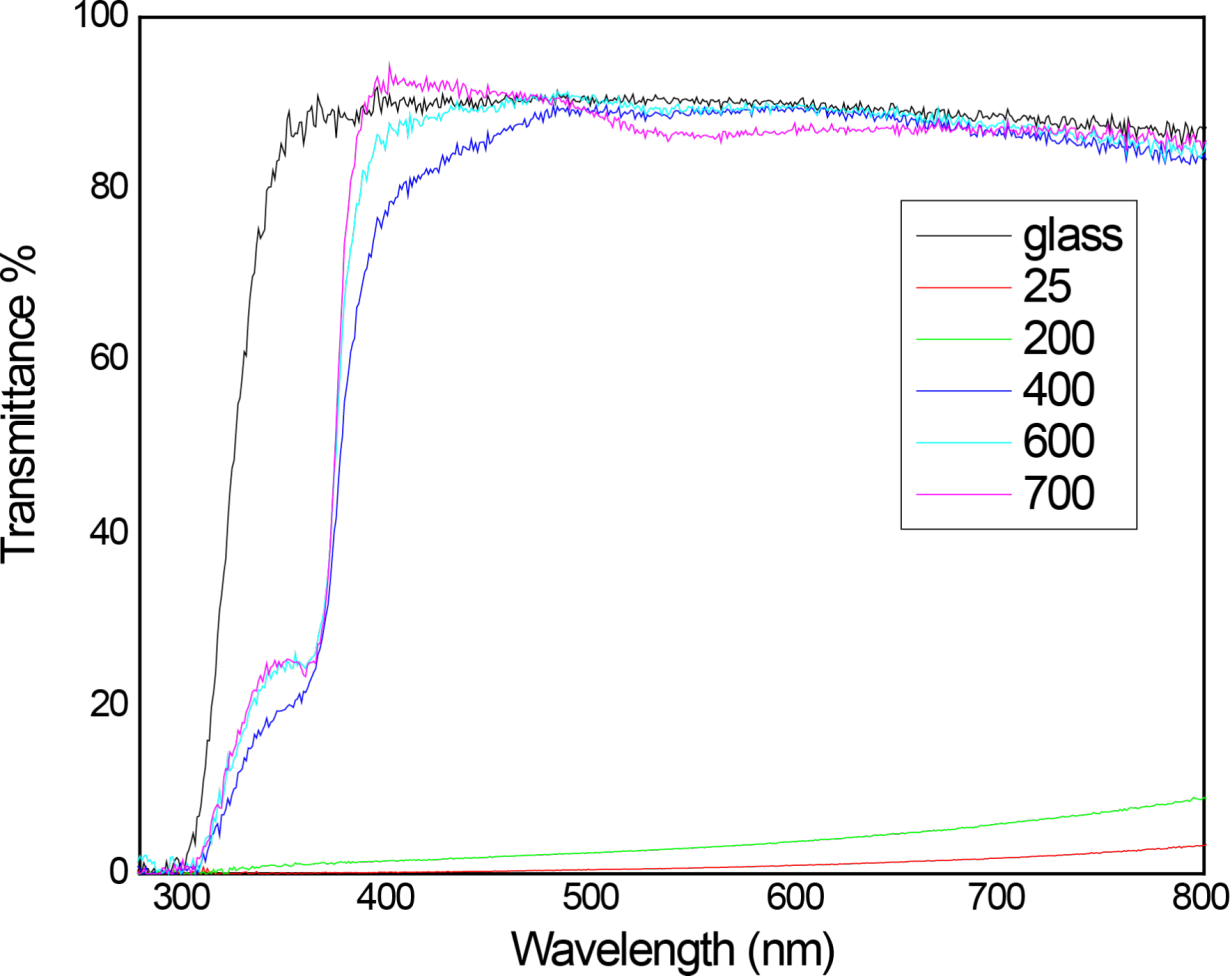
UV–vis spectra of ZnO thin films at different temperatures.

Optical characterization helps to trace the evolution and allow determining how the heat treatment affects the transmittance of the thin films. As it is shown in the transmittance spectra that the un-annealed films have a poor transmittance (less than 10%). However, the transmittance of the heat-treated films increases up to 80% (Figure 4). This difference is assumed to be due to the reorganization and coalescence of the nanoparticles and the formation of the worm-shape nanostructured thin films. In these films, a lot of space at the substrate is unoccupied by these nanostructures. Thus, it is assumed that the crystallization is improved when the temperature increases. The shoulder that appears in this spectrum is attributed to the porosity of this material and the spaces between the nanostructures; this is confirmed by the HRSEM images.

### Estimation of the optical band gap

Ignoring the reflectivity, which is expected to be low, the coefficient α may be determined from the results of the transmittance by using Equation 1:

[1]
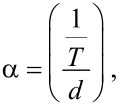


where *d* is the thickness of the thin film, and *T* is measured transmission. In consequence, the relation between the coefficient and the photon energy for direct transition is 
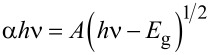
, where *A* is a constant, *E*_g_ is the optical band gap, the plot of this relation has a linear region, and the extrapolation of the straight line to zero gives the value of the energy gap of the film. The energy value obtained for this film is about 3.22 eV, and can be due to the grain boundaries and the size of the nanoparticles. As seen in the HRSEM images, there are a lot of grain boundaries and different sizes in the nanostructure. Film thicknesses between 45 nm and 123 nm were measured through profilometry after deposition times of 5, 10, 15, 20, and 25 min.

### Photoluminescence characteristics of thin films

**Figure 5:**
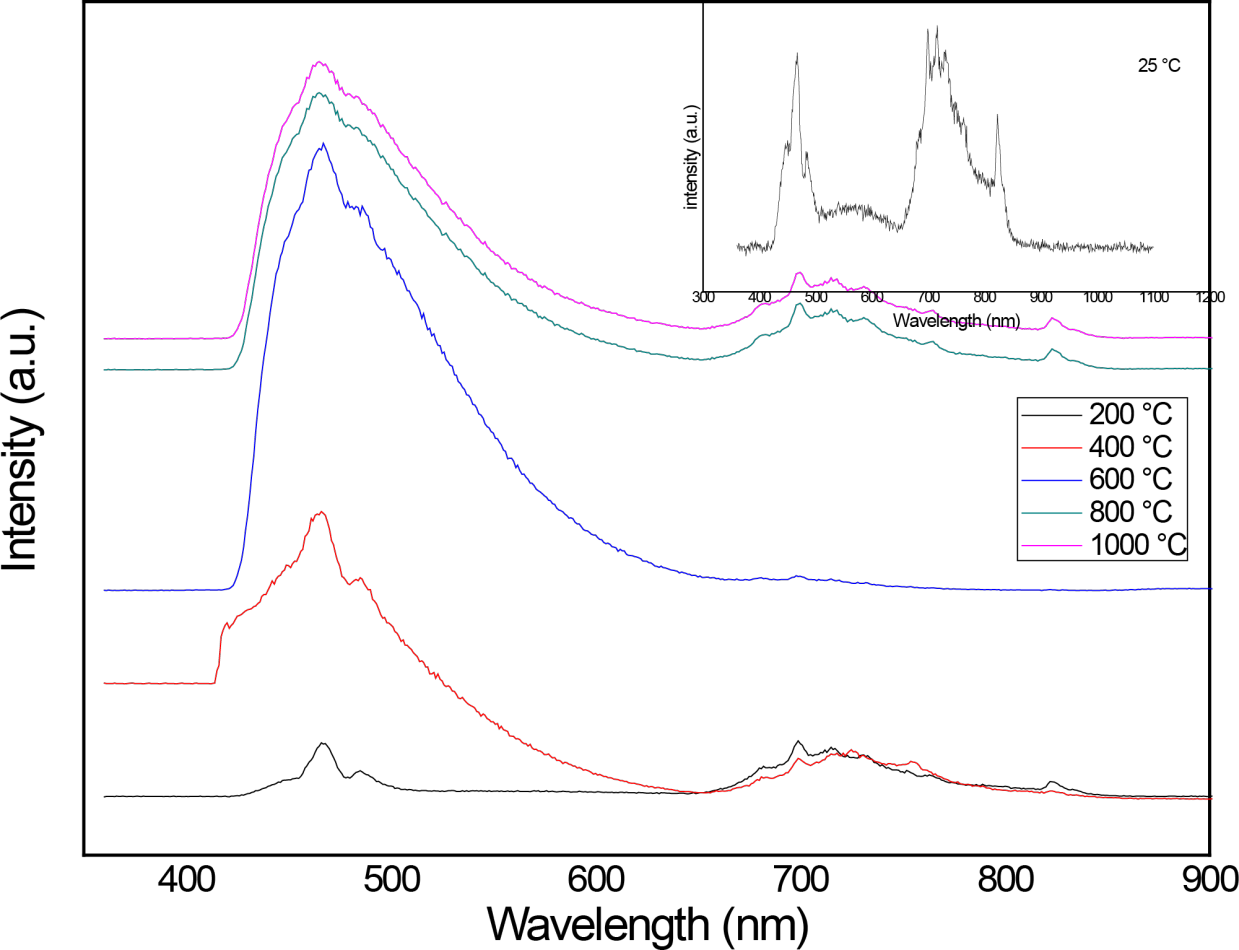
PL measurements of ZnO thin films annealed at different temperatures.

The ZnO thin films annealed at 25, 400 and 600°C were analyzed by PL shown in Figure 5. There are a lot of emission lines related to the characteristics of the deposited thin films, as the annealing temperature and the intrinsic defects (V_Zn_, V_O_). One of the emissions is in the blue region, which is close to 2.6 and 2.5 eV, and refers to the Zn vacancies. The emission close to 1.6 eV (red region) corresponds to oxygen vacancies (V_O_). The emission intensities are similar intensity for the thin films annealed at 400 and 25 °C. They change drastically when we have the nanostructures deposited as thin film where the main emission line attributed to V_Zn_ is related to the form of the nanostructured thin film and its distribution on the substrate. It is interesting to see the change in the intensity of the V_O_ contribution that decreases drastically and it is related to the formation of the nanostructures, as it is shown in the result obtained by PL compared with the HRSEM images.

## Conclusion

The authors successfully deposited worm-shaped nanostructured thin films by vacuum evaporation using colloidal nanoparticles as the source. The transmittance for annealed thin film is up to 80%, and this result is directly correlated with the annealing. The obtained thin films have a thickness between 45 nm and 123 nm by varying the deposition time (5 to 25 min). The XRD results show that this is a polycrystalline nanostructured thin film, and this is confirmed with the HRSEM images, but it is formed only at 600°C and disappears as the annealing temperature increases. From the photoluminescence we can check that the emission is in the blue and the red part of the visible light, due to the contribution of the V_O_, V_Zn_ and V_Zn_^−^ defects.
